# Hierarchical drug release designed Au @PDA-PEG-MTX NPs for targeted delivery to breast cancer with combined photothermal-chemotherapy

**DOI:** 10.1186/s12951-021-00883-8

**Published:** 2021-05-17

**Authors:** Wen Li, Zhiwen Cao, Liuchunyang Yu, Qingcai Huang, Dongjie Zhu, Cheng Lu, Aiping Lu, Yuanyan Liu

**Affiliations:** 1 School of Chinese Materia Medica, Beijing University of Chinese Medicine, Beijing, 100029 China; 2 Institute of Basic Research in Clinical Medicine, China Academy of Chinese Medical Sciences, Beijing, 100700 China; 3 School of Chinese Medicine, Hong Kong Baptist University, Kowloon, Hongkong, China

**Keywords:** Au @PDA-PEG-MTX NPs, Breast cancer targeted therapy, Fluorescence imaging, Hierarchical drug release, Combined photothermal-chemotherapy

## Abstract

Breast cancer (BC) is the most frequently diagnosed cancer with a low survival rate and one of the major causes of cancer-related death. Methotrexate (MTX) is an anti-tumor drug used in the treatment of BC. Poor dispersion in water and toxic side effects limit its clinical application. Gold nanoparticles (AuNPs), owing to their specific structures and unique biological and physiochemical properties, have emerged as potential vehicles for tumor targeting, bioimaging and cancer therapy. An innovative nano drug-loading system (Au @PDA-PEG-MTX NPs) was prepared for targeted treatment of BC. Au @PDA-PEG-MTX NPs under near infra-red region (NIR) irradiation showed effective photothermal therapy against MDA-MB-231 human BC cells growth in vitro by inducing apoptosis through triggering reactive oxygen species (ROS) overproduction and generating excessive heat. In vivo studies revealed deep penetration ability of Au @PDA-PEG-MTX NPs under NIR irradiation to find application in cancer-targeted fluorescence imaging, and exhibited effective photothermal therapy against BC xenograft growth by inducing apoptosis. Histopathological analysis, cellular uptake, cytotoxicity assay, and apoptosis experiments indicated that Au @PDA-PEG-MTX NPs possessed a good therapeutic effect with high biocompatibility and fewer side effects. This Au NPs drug-loading system achieved specific targeting of MTX to BC cells by surface functionalisation, fluorescence imaging under laser irradiation, combined photothermal-chemotherapy, and pH- and NIR- triggered hierarchical drug release.

**Supplementary Information:**

The online version contains supplementary material available at 10.1186/s12951-021-00883-8.

## Introduction

Breast cancer (BC) is one of the most frequent malignancy in women, and is associated with a high mortality rate and economic burden [1, 2]. Owing to its complicated etiology, poor response and severe side-effects of chemotherapy, both safety and effectiveness are considered as the key challenges to prevent deaths [3]. The main aim is to develop effective therapeutic strategies with low toxicity and high specificity to eliminate tumors in the fight against BC [4]. However, currently used BC treatment approaches, such as surgery, chemotherapy, and radiotherapy, cause diverse side effects in patients, and hence these approaches alone are unable to achieve the aim [5]. The combination of chemotherapeutic drugs and the gold nanoparticles (AuNPs) carrier system with photothermal property can provide a promising platform for intracellular delivery of various anti-BC drugs and synergistic therapy [6, 7].

Targeted NPs designed for cancer treatment can deliver chemotherapeutic drugs to specific cancer cells while reducing the exposure of normal healthy cells; therefore, larger doses of drugs can be delivered to the tumor site to achieve therapeutic effects with high targeting and low toxicity [8]. AuNPs are a type of inorganic cargo with versatile surface for multi-functionalization and large surface area-to-volume ratio for drug loading, and possess superior optical properties for bioimaging and even photothermal properties for therapy; however, toxicity and low biocompatibility of AuNPs are the challenges that cannot be ignored [9]. Optimization of drug delivery can be achieved by adjusting the size and shape of AuNPs [10, 11]; small-sized of AuNPs (15 nm of diameter) can be used to reduce its toxicity attributable to being an inorganic material.

Interestingly, AuNPs have attracted great attention during the past decade owing to their facile synthesis and surface functionalization [12], along with high photothermal conversion capacity in the near infrared region (NIR) without harmful side effects in biological systems [13–15]. AuNPs can be used in NIR imaging of tumors, as they possess good photostability [16]. In addition, the photothermal conversion produces excessive heat and reactive oxygen species (ROS) that destroys cancer cells [17]. To make AuNPs an ideal drug carrier, they should be modified to achieve targeted delivery and controlled release [18]. The strong adhesion of polydopamine (PDA) is conductive to its deposition on AuNPs, which can improve the drug loading capacity and biocompatibility, and AuNPs internalisation by mammalian cells [19]. Moreover, PDA shell can prevent the leakage of loaded drugs during delivery, simultaneously achieving an on-demand drug release in the targeted location, such as NIR stimuli responsive drug release under high temperature or acidic conditions [19–21]. Besides, PEG can also improve cellular uptake of gold nanocomposites and prolong the plasma circulation time [22–24]. In addition, drugs linked to PEG are released under acidic conditions, which can lead to development of a hierarchical drug release system [25].

Methotrexate (MTX), a folic acid analogue, is a highly potent inhibitor of the folate pathway that induces cell apoptosis, and is widely used for the treatment of rheumatoid arthritis and acute leukemia [26]. Owing to its special characteristic, many experimental studies have explored its use in the treatment of cancer [27]. MTX can inhibit dihydrofolate reductase enzyme that helps produce tetrahydrofolate and its by-products, which are essential for the growth of tumor cells [28, 29]. Folate receptor (FR) is a glycosyl phosphatidylinositol-linked protein has low expression in normal tissues, but is overexpressed in certain malignant cells such as those of BC [30]. MTX exhibits high affinity for FR, and therefore can be targeted to BC cells, in addition to its therapeutic use being folate analogue. However, similar to most of the conventional chemotherapeutic drugs, MTX is associated with toxin accumulation in the body, accompanied by low specificity and poor therapeutic efficacy, the main challenges of single-agent chemotherapy [31]. Therefore, in recent years, therapeutic strategy against BC primarily includes the combination of chemotherapeutics and nanomaterials [32]. Here, MTX loaded in AuNPs is not only a combination of photothermal therapy and chemotherapeutic drug, but can be precisely identified as targeted ligands for receptors highly expressed on BC cell surface.

**Fig. 1:**
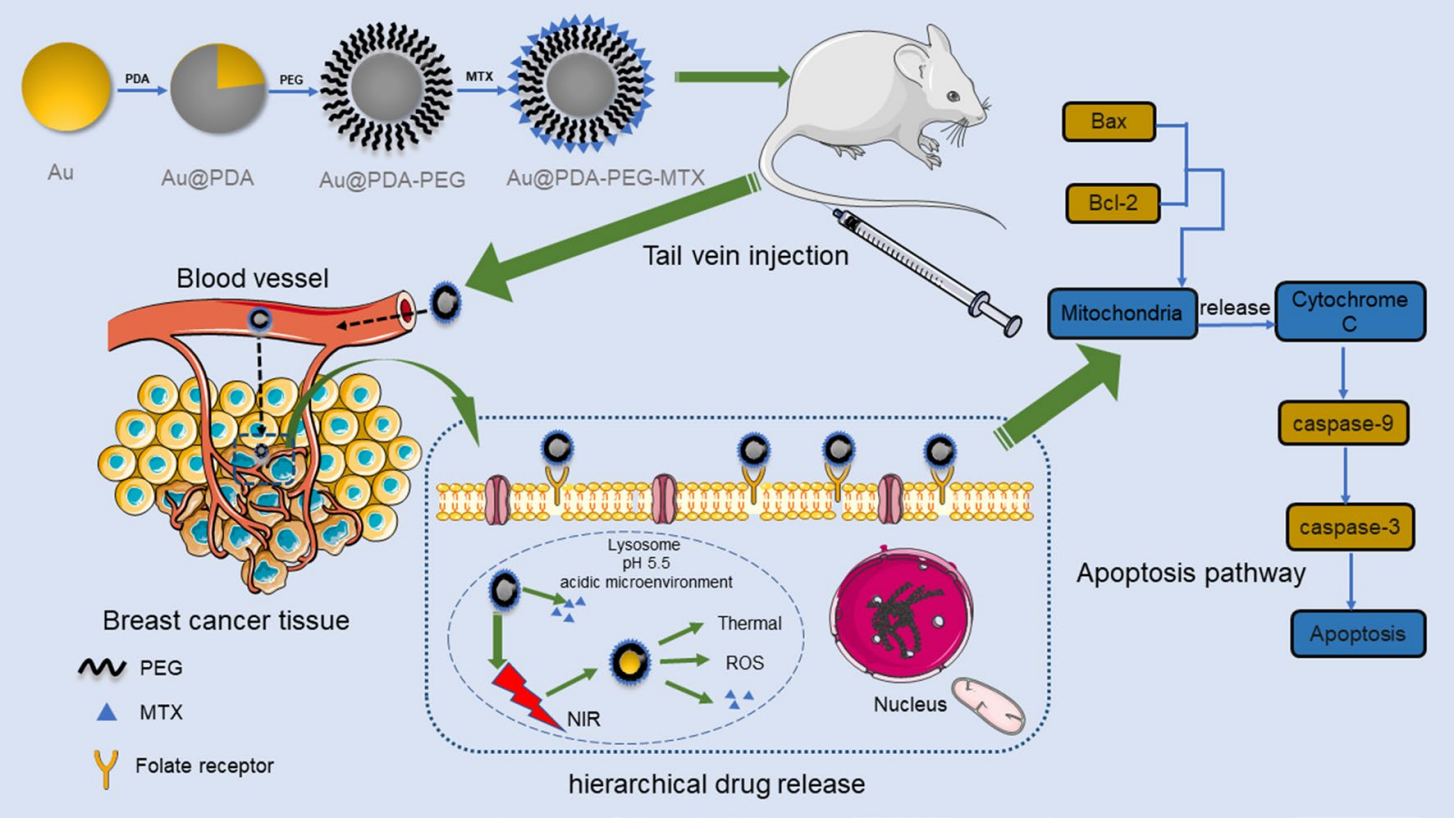
Process diagram for synthesis of Au @PDA-PEG-MTX, targeting tumor tissues in vivo, releasing drugs and pathways that cause apoptosis

In the present study, Au @PDA-PEG-MTX NPs were designed for targeted delivery and hierarchical release in combination of photothermal therapy and chemotherapy for BC with improved efficiency and low toxicity. In this novel nanodrug delivery system, AuNPs not only act as drug carriers; additionally, when modified with PDA and PEG, they exhibit synergistic chemo-photothermal therapeutic efficacy, bioimaging, and NIR- and acidic conditions-stimulated hierarchical release (Fig. 1).

## Methods

### Reagents

The following chemicals were used without further purification and purchased from commercial sources as follows: chloroauric acid (99.95%) from Innochem (Beijing, China); dopamine hydrochloride, 1-ethyl-3-(3-dimethylaminopropyl) carbodiimide hydrochloride (EDCI), N-hydroxy succinimide (NHS) and dimethyl sulfoxide (DMSO) from Sigma-Aldrich (St Louis, MO, USA); and 2ʹ,7ʹ-dichlorofluorescin diacetate (DCFH-DA) from Beyotime Biotechnology (Shanghai, China). All aqueous solutions were prepared using double-distilled water. Foetal bovine serum (FBS) and Dulbecco’s Modified Eagle Medium (DMEM)/High Glucose medium were purchased from Gibco (Waltham, MA, USA). Penicillin-Streptomycin (penicillin 100 U/mL and streptomycin 100 mg/mL) were obtained from BI(IL). Human breast cancer MDA-MB-231 cells were obtained from BNCC (Beijing, China). All antibodies were purchased from Abcam (Camb, UK).

### Preparation of Au @PDA-PEG-MTX NPs

#### Preparation of AuNPs

AuNPs (15 nm in diameter) were synthesized according to the citrate reduction method reported by Frens [33]. Before the start of reaction, the reaction vessel was thoroughly washed with freshly prepared aqua regia (HNO_3_/HCl = 1:3) and then washed three times with double distilled water. First, 1 mL of 1% chloroauric acid solution was added to 100 mL of ultrapure water, and was quickly boiled. Then, 4 mL of 1% sodium citrate solution was added immediately. When the color of the solution changed from light yellow to wine red, AuNPs with an average diameter of 15 nm were formed, which were stored under dark conditions at 4 °C. The average size of the AuNPs was estimated using transmission electron microscopy (TEM, HT7700, Tokyo, Japan, Hitachi).

#### Preparation of Au @PDA NPs bioconjugates

Previously synthesized AuNPs were dispersed in 50 mL of Tris-HCl buffer (pH 8.5) at a concentration of 2 mg/mL. The solution was subjected to magnetic stirring at high speed overnight in the dark, and then 5 mL (at a concentration of 2 mg/mL) of the prepared dopamine hydrochloride solution was added dropwise to this. A solution black in color was obtained, which was centrifuged at 12,000 rpm at 4 °C for 10 min; the supernatant was discarded, and the black precipitate was collected and washed three times with deionized water.

#### Preparation of Au @PDA-PEG NPs

The synthesized Au @PDA NPs complex was dispersed in 50 mL of deionized water at a concentration of 2 mg/mL. NH_2_-PEG-SH (molecular weight 2k) (at a concentration of 2 mg/mL) was added dropwise to the solution and magnetically stirred overnight in the dark. Then, the solution was centrifuged at 12,000 rpm at 4 °C for 10 min to obtain Au @PDA-PEG NPs, which were washed three times with deionized water [34].

#### Preparation of Au @PDA-PEG-MTX NPs

To load MTX on the synthesized Au @PDA-PEG NPs, 8 mg MTX, 4 mg EDCI, and 2.8 mg NHS were added to 50 mL aqueous solution of Au @PDA-PEG NPs under magnetic stirring for 3 h in the dark; the mixture was centrifuged at high speed at 4 °C (12,000 rpm, 10 min), the supernatant was saved, and the precipitate was washed three times with deionized water. The precipitate was the Au @PDA-PEG-MTX NPs bioconjugate.

#### Preparation of Au @PDA/FITC-PEG-MTX NPs

The synthesized Au @PDA-PEG-MTX NP bioconjugate was re-dispersed in 2 mL of deionized water (at a concentration of 2 mg/mL). Next, the solution was magnetically stirred continuously for 12 h at room temperature in the dark, and 50 µL of DMSO solution containing 10% (w/w) fluorescein isothiocyanate (FITC, fluorescent agent) was added. The resulting mixture was then centrifuged at 4 °C (12,000 rpm, 10 min), and the precipitate obtained after centrifugation was washed three times with deionized water. The final product obtained was Au @PDA/FITC-PEG-MTX NPs. Same procedure was followed to synthesize Au @PDA/cyanine7 (Cy7)-PEG-MTX NPs.

### Drug loading study of Au @PDA-PEG-MTX NPs

Ultraviolet-visible (UV-vis) spectroscopy was used to determine MTX loading efficiency by measuring the absorbance of the drug after washing, supernatant, and the initial drug. MTX loading efficiency was calculated as follows:

$$\text{Loading efficiency} \,(\%)=\frac{(A_{Drug}-A_s{-A}_w)}{A_{Drug}}\times100\%$$

*A*_*W*_ is absorbance of the drug after washing, *A*_*S*_ is absorbance of supernatant and *A*_*Drug*_ is absorbance of initial drug [30].

### In vitro MTX release pattern from Au @PDA-PEG-MTX NPs

Au @PDA-PEG-MTX NP bioconjugate was dissolved in 10 mL phosphate buffer solution (20 mM) at pH 5.4 and 7.4, and the solution was placed in a prepared dialysis bag with a molecular weight cut-off of 1 kDa. Simultaneously, protease (1 mg/mL) was added to phosphate buffer to cleave the amide bond between MTX and PEG [35]. At the specified time point, 500 µL of the dialyzed solution was collected to measure the released MTX, and the absorbance was measured at 305 nm by UV-vis spectroscopy (UV-vis, PerkinElmer, Singapore) [36].

### Characterization of Au @PDA-PEG-MTX NPs and its intermediate products

A UV-vis spectrometer was used to record the UV-vis absorption spectrum of the synthesized AuNPs (15 nm in diameter). A Fourier transform infrared spectrometer (FT-IR, Thermo Fisher Scientific, USA) was used to record the infrared spectrum of MTX-PEG. The morphology of AuNPs, Au @PDA NPs and Au @PDA-PEG-MTX NPs were observed by TEM. The Brookhaven Zeta PALS instrument was used to record dynamic light scattering (DLS) intensity and zeta potential of AuNPs, Au @PDA NPs, Au @PDA-PEG NPs and Au @PDA-PEG-MTX NPs.

### Biological experiments

#### Cell culture and in vitro cytotoxicity assay

Human BC MDA-MB-231 cells were cultured in a high glucose medium containing 10% FBS, and incubated at 37 °C in a 5% CO_2_ atmosphere [37]. MDA-MB-231 cells were digested with trypsin containing EDTA, and the digested MDA-MB-231 cells were seeded in a transparent 96-well plate. The cell density of each well was 1.8 × 105 cells, which were incubated overnight in an incubator to allow cell attachment. Further, 2.5–30 µg/mL of Au @PDA-PEG-MTX NPs were added for 12 h, and the cells were irradiated with or without 808 nm NIR (200 mW/cm^2^) (Taizhu Anford Laser, China) for 20 min. Cell Counting Kit-8 (CCK-8) was used to measure and evaluate the cell viability. A microplate reader (Molecular Devices Tecan M200 PRO) was used to measure the absorbance at 450 nm. The cell survival rate (%) was calculated as (average absorbance value of the treatment group/average absorbance value of the control group) ×100%. The measurements were repeated three times.

#### In vitro cellular uptake of Au @PDA-PEG-MTX NPs

The uptake of Au @PDA/FITC-PEG-MTX NPs by MDA-MB-231 cells was qualitatively recorded using a confocal laser scanning microscope. MDA-MB-231 cells were digested with trypsin, and then 1 mL of digested MDA-MB-231 cells (1 × 10^5^) were inoculated into a glass-bottom cell culture dish and incubated in an incubator for 24 h. The original medium was removed and the cells were washed with phosphate-buffered saline (PBS). Nanocomposite Au @PDA/FITC-PEG-MTX was used at concentrations of 15 µg/mL, 20 µg/mL and 25 µg/mL to replace this medium and incubated for 4 h to completely absorb the drug. The cells were washed three times with PBS and fixed with 4% cell fixation solution (1 mL/well) at 4 °C for 30 min. The cells were again washed three times with PBS, and then stained with diamidino-2-phenylindole (DAPI) solution (2 mL/well) for 5 min to ensure coloration of the nuclei. After washing the cells with PBS, confocal laser scanning imaging was performed.

The uptake of Au @PDA/FITC-PEG-MTX NPs by MDA-MB-231 cells was quantificationally recorded using a flow cytometer (FCM, CytoFLEX, USA). MDA-MB-231 cells were planted in a six-well plate at a density of 1 × 10^6^ cells per well and incubated in an incubator for 24 h. Au @PDA/FITC-PEG-MTX was used at concentrations of 15 µg/mL, 20 µg/mL and 25 µg/mL to replace this medium and incubated for 4 h to completely absorb the drug. Set MDA-MB-231 cells without drugs as the negative control group. The digestion solution was added to each well of the six-well plates, and a large amount of PBS was added after the digestion to stop the digestion. Centrifuge the mixture in a centrifuge tube, remove the supernatant, and add a large amount of PBS to repeat the above operation. After the cells were washed, 1 mL PBS was added to prepare a suspension, and then transferred to a clean glass test tube for fluorescence intensity testing by FCM.

#### Measurement of ROS generation

MDA-MB-231 cells were seeded in 6-well plates (10^5^ cells/well) and divided into three groups: blank cells, Au @PDA-PEG-MTX NPs and NIR + Au @PDA-PEG NPs. The cells were incubated for 24 h in a cell culture incubator. The NIR + Au @PDA-PEG NPs group used 808 nm NIR laser radiation to treat the cells. DCFH-DA probe was diluted 1:1000 in serum-free medium; the medium containing DCFH-DA probe was added to each well to cover the cells, and then incubated in an incubator for 30 min. The cells were then washed with PBS, and a laser confocal microscope was used to set the excitation wavelength to 488 nm and the emission wavelength to 525 nm to detect the generation of ROS.

#### Western blot analysis

The cells were treated with MTX, Au @PDA-PEG-MTX NPs and NIR + Au @PDA-PEG-MTX NPs and total protein was extracted and quantified using the bicinchoninic acid (BCA) kit. Western blotting was used to analyse the effects of MTX, Au @PDA-PEG-MTX NPs and NIR + Au @PDA-PEG-MTX NPs on the expression of Caspase 3, Caspase 9, Bcl-2 and Bax proteins involved in the apoptotic pathway.

### In vivo distribution

BALB/c female nude mice were purchased from Beijing Vital River Laboratory Animal Technology Co., Ltd. and bred in a sterile environment (SPF). Four-week-old BALB/c nude mice were used as experimental subjects. The experimental nude mice were anesthetized by intraperitoneal injection of 300 µL of 2% sodium pentobarbital (215 mg/kg). Place the nude mouse in the prone position in the recording dark box of the small animal multispectral live imaging system. BALB/c female nude mice were administered 2 mg/kg Au @PDA/Cy7-PEG-MTX NPs intravenously via tail injections. The nude mice in the control group were injected with Cy7. The fluorescence images of the nude mice were detected at 0, 2, 6, 12, 24 and 48 h after injection using the IVIS Spectrum (Carestream Health Fx Pro/FX) in vivo fluorescence imaging system. The excitation wavelength was set at 743 nm and the emission wavelength was set at 767 nm for Au @PDA/Cy7-PEG-MTX NPs.

### In vivo anticancer effects

The tumor model was established by subcutaneously injecting MDA-MB-231 cells (10^6^ cells in 100 µL) into the armpit of nude mice. After 16 days of inoculation, the tumor volume reached 90 mm^3^. The mice were randomly divided into four groups, each with 6 mice: saline control group, MTX treatment group, Au @PDA-PEG-MTX NPs treatment group, and NIR + Au @PDA-PEG-MTX NPs treatment group. The mice were given 2 mg/kg Au @PDA-PEG-MTX NPs intravenously via tail injection, and the weights of the mice were recorded in real time. After 19 days of treatment, the mice were sacrificed, and th**e** tumor mass was surgically removed to measure the volume and weight. The tumor inhibitory rate was as follows:

$$\text{Tumor inhibitory rate}\,(\%)=\frac{W_c-W_o}{W_c}\times100{\%}$$

*W*_c_ is the average tumor weight of the control group, and *W*_o_ is the average tumor weight of the operation group.

### Safety evaluation of Au @PDA-PEG-MTX NPs

To evaluate in vivo toxicity of Au @PDA-PEG-MTX NP and NIR + Au @PDA-PEG-MTX NPs, healthy BALB/c mice were set as the control group, and BALB/c nude mice were sacrificed 30 days after administration of drug. The important organs of mice (liver, spleen, kidney, heart and lung) were collected and stained with haematoxylin and eosin (H&E) stain to observe histopathological changes.

### Statistical analysis

All data and images were from three independent experiments. Data were expressed as mean ± SD. Statistical analysis was performed by Prism graph pad 8.0, and then Tukey’s post-test was performed. *P < 0.05 vs. blank, **P < 0.01 vs. blank, ***P < 0.001 vs. blank, ****P < 0.0001 vs. blank.

## Results and discussion

### Characterization of Au @PDA-PEG-MTX NPs and its intermediate products

**Fig. 2:**
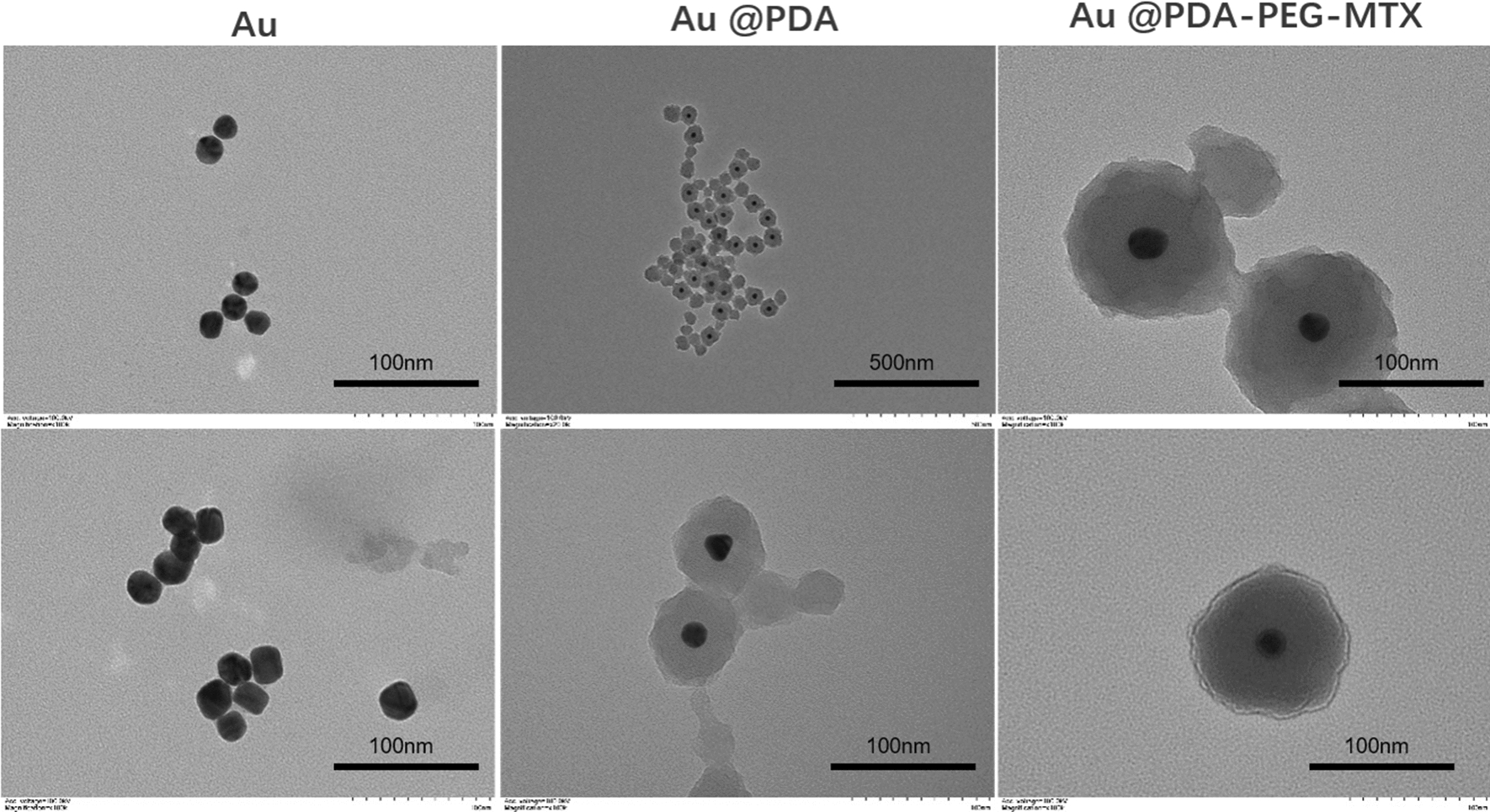
From left to right were the morphological characteristics of the synthesized Au, Au @PDA and Au @PDA-PEG-MTX by TEM

**Fig. 3:**
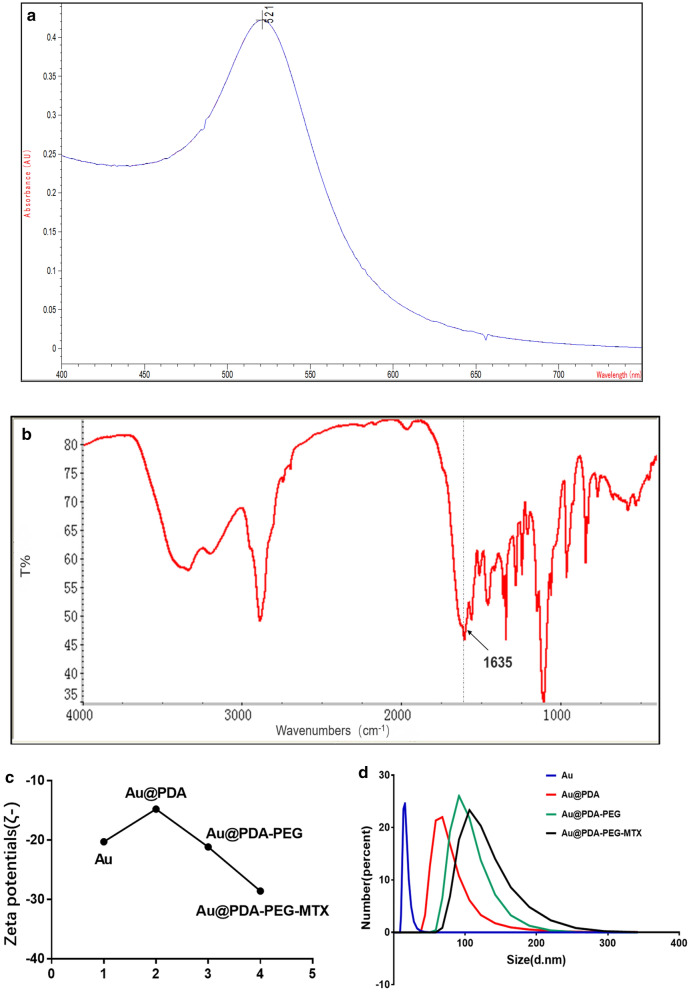
**a** The image of UV-vis spectrum showing a characteristic absorption peak of AuNPs at 521 nm; **b **FT-IR spectrum showing the absorption peak of the infrared spectrum at 1635 cm^− 1^ is significantly enhanced; **c **Value changes of Au, Au @PDA, Au @PDA-PEG and Au @PDA-PEG-MTX zeta potential; **d** Size distribution of Au, Au @PDA, Au @PDA-PEG and Au @PDA-PEG-MTX

Uniform-sized AuNPs having approximately 15 nm diameter were prepared by citrate reduction method, as validated by TEM analysis (Fig. 2). Dopamine was polymerized spontaneously in an alkaline environment and adsorbed on the surface of AuNPs to form PDA. The shape of Au @PDA NPs was shown in Fig. 2. The maximum absorption peak in the ultraviolet spectrum was 521 nm, indicating the synthesis of AuNPs once again (Fig. 3a). The structure of MTX-PEG was identified by infrared spectroscopy and further characterisation was done after synthesis. As shown in Fig. 3b, the absorption peak of the infrared spectrum at 1635 cm^−1^, which was related to the stretching vibration of amide (C=O), was significantly enhanced after synthesis suggesting formation of a new amide bond and further explained the synthesis of MTX-PEG. The zeta potential measurement at each step in the synthesis process from Au NP to Au @PDA-PEG-MTX NP indicated that the modification was successfully carried out at each step of the process. Zeta potential of AuNP prepared by the citrate reduction method was negative at -20.3 mV. Au @PDA NPs were synthesised using dopamine hydrochloride, which ionized hydrogen ions in an aqueous solution, as indicated by the zeta value of − 14.8 mV. Au @PDA-PEG NPs were obtained by Michael addition reaction. The negative ions produced by this reaction changed the zeta potential to − 21.2 mV. Au @PDA-PEG-MTX NPs were obtained through an amidation reaction. This reaction further reduced the positive charge by blocking the amino group and not ionising the positively charged elements. The final zeta potential value of Au @PDA-PEG-MTX NP was measured at − 28.6 mV (Fig. 3c). AuNPs, Au @PDA NPs, Au @PDA-PEG NPs and Au @PDA-PEG-MTX NPs showed excellent dispersibility in solution. As shown in Fig. 3d for the measurement results of dynamic light scattering (DLS), the particle size distribution of AuNPs, Au @PDA NPs, Au @PDA-PEG NPs and Au @PDA-PEG-MTX NPs measured through DLS instrument were 16.6, 126.2, 138.4 and 154.9 nm respectively.

AuNPs were one of the most used inorganic nanocarriers. Under NIR irradiation, these nanocarriers generated heat and ROS to promote cell apoptosis. AuNPs having 15 nm diameter have been experimentally proven to be less toxic than other nanoparticles, and showed better tissue penetration, escaped recognition and clearance in the blood circulation [23]. PDA had low toxicity and was easily soluble in water, which can improve the toxicity and biocompatibility of the gold nanocomposites. In addition, PDA was absorbed in the near-infrared light region and disintegrated under high temperature or acidic conditions to allow drug release, making it a potential photothermal therapeutic agent. To improve the dispersibility and biocompatibility of AuNPs in aqueous solution, NH_2_-PEG-SH (2k) was used. The amino group of NH2-PEG-SH(2k) was conjugated to the targeting drug MTX through amidation reaction. The sulfydryl group of NH2-PEG-SH(2k) was linked to the PDA adhering to the surface of the AuNPs through the Michael addition reaction. Experiments have proved that PEG could be used to functionalize Au and modify the surface of Au to facilitate drug loading and reduce toxicity. Moreover, PEG could improve cellular uptake of gold nanocomposites. MTX, a commonly used anti-BC drug, caused certain toxicity to normal tissues during metabolism due to the production of various metabolites, which was also a tumor-targeting ligand. The Au @PDA-PEG-MTX NPs exhibited good biocompatibility, dispersibility and tumor-targeting effect.

### Drug loading and release

MTX loading efficiency was determined by comparing the absorbance of the supernatant obtained by centrifuging the drug-loaded particles and the initial drug in the drug-loading experiment. MTX was an anti-BC drug that had severe side effects. In this study, the carboxyl group of MTX was coupled with amine-functionalised polyethylene glycol to reduce toxicity and improve its effect against BC. The loading efficiency of MTX was observed to be 36.21% at drug-particles ratio of 3:1.

**Fig. 4:**
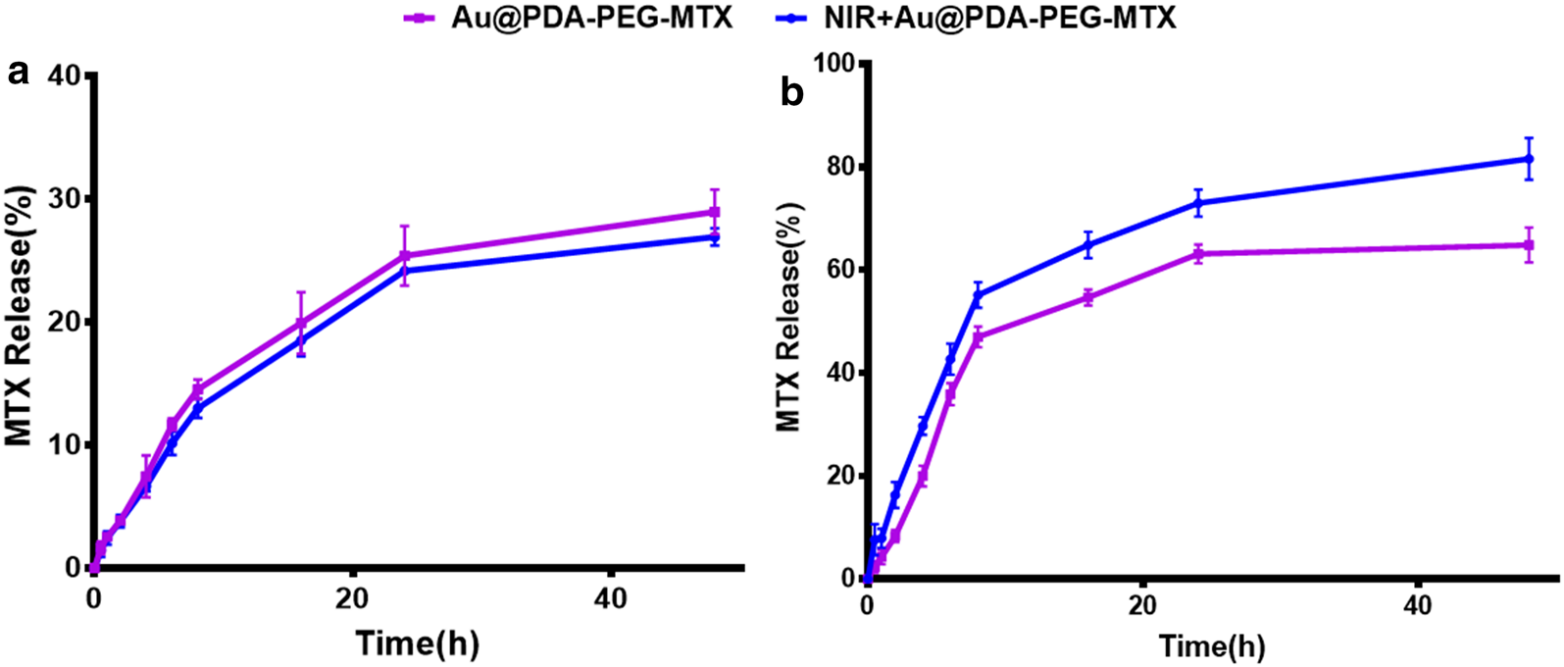
**a** Release of MTX by nanocomposites when Au @PDA-PEG-MTX placed in the simulated normal physiological environment pH 7.4; **b** Release of MTX by nanocomposites when Au @PDA-PEG-MTX placed in the simulated tumor acid environment pH 5.5; MTX released by NIR irradiation with 808 nm (blue curve) and MTX released by nanometers not irradiated by 808 nm NIR (pink curve)

To evaluate MTX release from Au @PDA-PEG-MTX NPs in normal physiological (pH 7.4) environment and tumor lysosomal (pH 5.5) microenvironment in the body, UV spectrophotometry was used for quantification. PBS buffer was used as the simulated body fluid. Figure 4 showed the release of MTX at pH 5.5 and pH 7.4 after 48 h. The release of MTX from Au @PDA-PEG-MTX NPs was observed to be pH-dependent. At pH 5.5, MTX was released rapidly, with a cumulative release of 64.83% within 60 h. This release might be attributable to the hydrolysis of the amide bond connected to MTX to release a large amount of drugs under the acidic conditions of the simulated lysosomal environment. According to Fig. 4, the release of MTX at pH 7.4 after 48 h was 28.97%, while using 808 nm NIR laser to irradiate Au @PDA-PEG-MTX NPs solution, the release percentage of MTX was 26.93%; this might be because the amide bond is relatively stable under normal physiological conditions. In addition, the release of MTX from Au @PDA-PEG-MTX NPs irradiated with NIR laser was studied. At pH 5.5, cumulative release of MTX reached to 64.83% within 48 h vs. 81.56% for 808 nm NIR laser irradiation, which was much higher than that of MTX without NIR laser irradiation under the same conditions. In comparison, MTX was hardly released in the normal physiological environment (pH 7.4) neither with or without NIR laser irradiation. This showed that Au @PDA-PEG-MTX NPs were relatively stable under normal physiological conditions and performed on pH-dependent and NIR laser-irradiated drug release. We speculated that besides being bonded (amide bonds) to the gold nanocomposite, MTX might have adsorbed on the surface of the gold nanocomposite through its adhesion to PDA.

### Cytotoxicity of nanocomposite

**Fig. 5:**
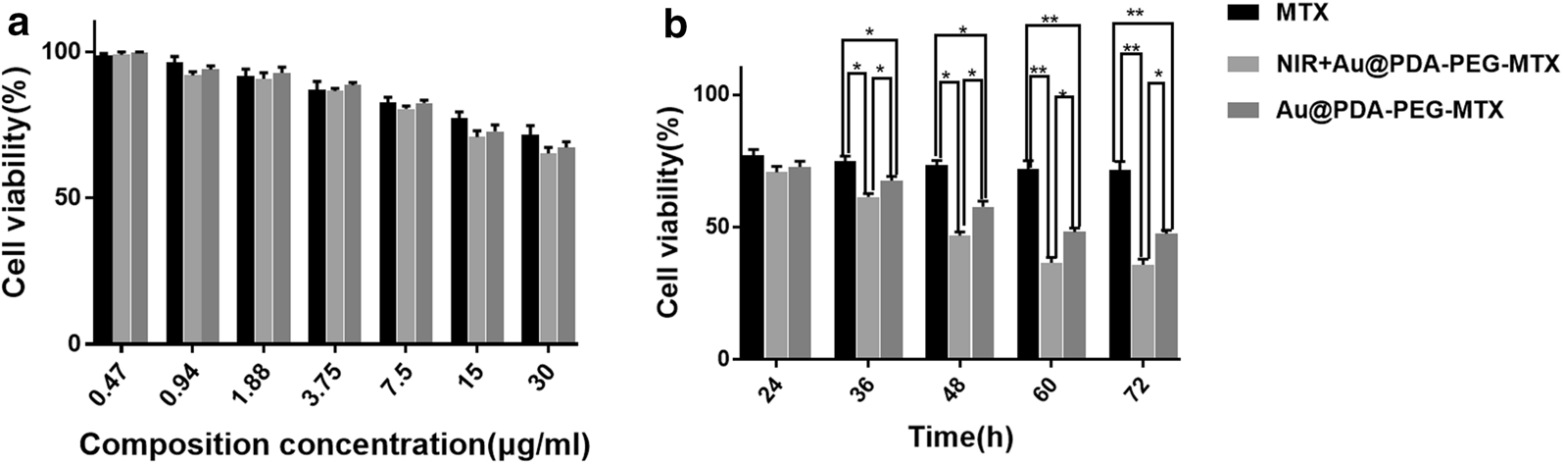
**a **The cytotoxicity of MTX, Au @PDA-PEG-MTX and NIR + Au @PDA-PEG-MTX against MDA-MB-231 cells; **b** The cytotoxicity of MTX, Au @PDA-PEG-MTX and NIR + Au @PDA-PEG-MTX against MDA-MB-231 cells after 36, 48, 60 and 72 h when controlling the dosage at 15 µg/mL (*p < 0.05, **p < 0.01)

The cytotoxicity of Au @PDA-PEG-MTX NPs was examined in MDA-MB-231 cells using the CCK-8 assay. The cell viability experiment in the present study was to screen the appropriate concentration of MTX, NIR + Au @PDA-PEG-MTX and Au @PDA-PEG-MTX with concentration-dependent cytotoxicity in MDA-MB-231 cells within 24 h. As shown in Fig. 5 A, MDA-MB-231 cells were treated with different concentrations of MTX, Au @PDA-PEG-MTX NPs and NIR + Au @PDA-PEG-MTX NPs. The effect of NIR or nanoformulations on cytotoxicity needs a release process. The reason why the nanoformulations seem rather ineffective may be that it was performed at 24 h, for which nanoformulations were not adequately released.

The cytotoxicity of NIR or nanoformulations at 15–30 µg/mL changed significantly compared to the previous concentration. Therefore, 15–30 µg/mL was selected in subsequent experiments. Furthermore, cell viability of MDA-MB-231 cells incubated with 15 µg/mL MTX, NIR + Au @PDA-PEG-MTX and Au @PDA-PEG-MTX after 24, 36, 48, 60 and 72 h could be provided in Fig. 5b. Before 24 h, the three groups showed no significant differences due to little drug release. During 36–60 h, the nanoformulations were released (Fig. 4b), then significant differences between MTX vs. Au @PDA-PEG-MTX, MTX vs. NIR + Au @PDA-PEG-MTX, and even Au @PDA-PEG-MTX vs. NIR + Au @PDA-PEG-MTX were observed. It showed that the toxicity of Au @PDA-PEG-MTX was greater than the pure MTX, whilst, NIR + Au @PDA-PEG-MTX was greater than that of Au @PDA-PEG-MTX against MDA-MB-231 cells after 36 h.

### Cellular uptake of Au @PDA-PEG-MTX NPs

The degree to which the nanomedicine was taken up by the cells affected the delivery and therapeutic effect of the drug. This was demonstrated by confocal fluorescence microscopy, in which the synthesized nanocomposite, Au @PDA-PEG-MTX NPs were labelled with FITC. After MDA-MB-231 cells were incubated with Au @PDA/FITC-PEG-MTX NPs, confocal laser scanning imaging showed strong fluorescence signals of Au @PDA/FITC-PEG-MTX NPs in the cytoplasm. Meanwhile, FCM was used to quantify the cellular uptake.

**Fig. 6:**
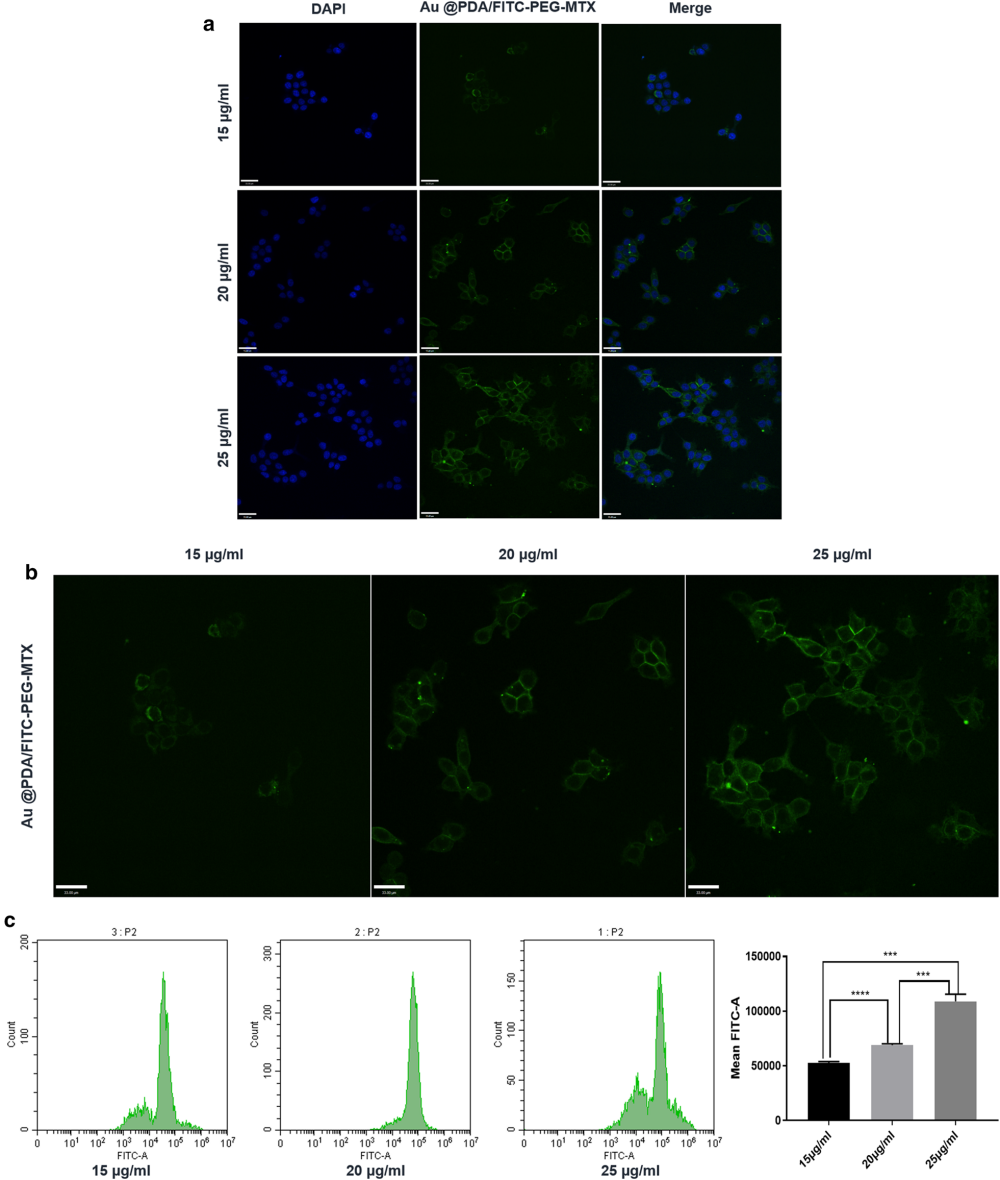
**a** CLSM images of MDA-MB-231 cells incubated with 15 µg/mL, 20 µg/mL, 25 µg/mL Au @PDA-PEG-MTX; **b** Single channel image of the uptake of 15 µg/mL, 20 µg/mL and 25 µg/mL Au @PDA/FITC-PEG-MTX; **c** Quantitative calculation of the uptake of Au @PDA/FITC-PEG-MTX through FCM (***p < 0.001, ****p < 0.0001)

The results suggested that the Au @PDA-PEG-MTX NPs can be efficiently internalized by human BC MDA-MB-231 cells. In addition, different fluorescence intensities indicating different cellular uptake could be observed at different dosages, with strongest fluorescence intensity at 25 µg/mL, indicating that the drug at this concentration was maximally taken up by the cells (Fig. 6).

### ROS detection in MDA-MB-231 cells

Photothermal therapy can cause ROS production. According to recent studies, ROS can accelerate tumor cell death, which is considered as the main mechanism of photothermal therapy against cancer. Therefore, a laser confocal microscope was used to detect ROS generation in cells treated with NIR + Au @PDA-PEG-MTX NPs.

**Fig. 7:**
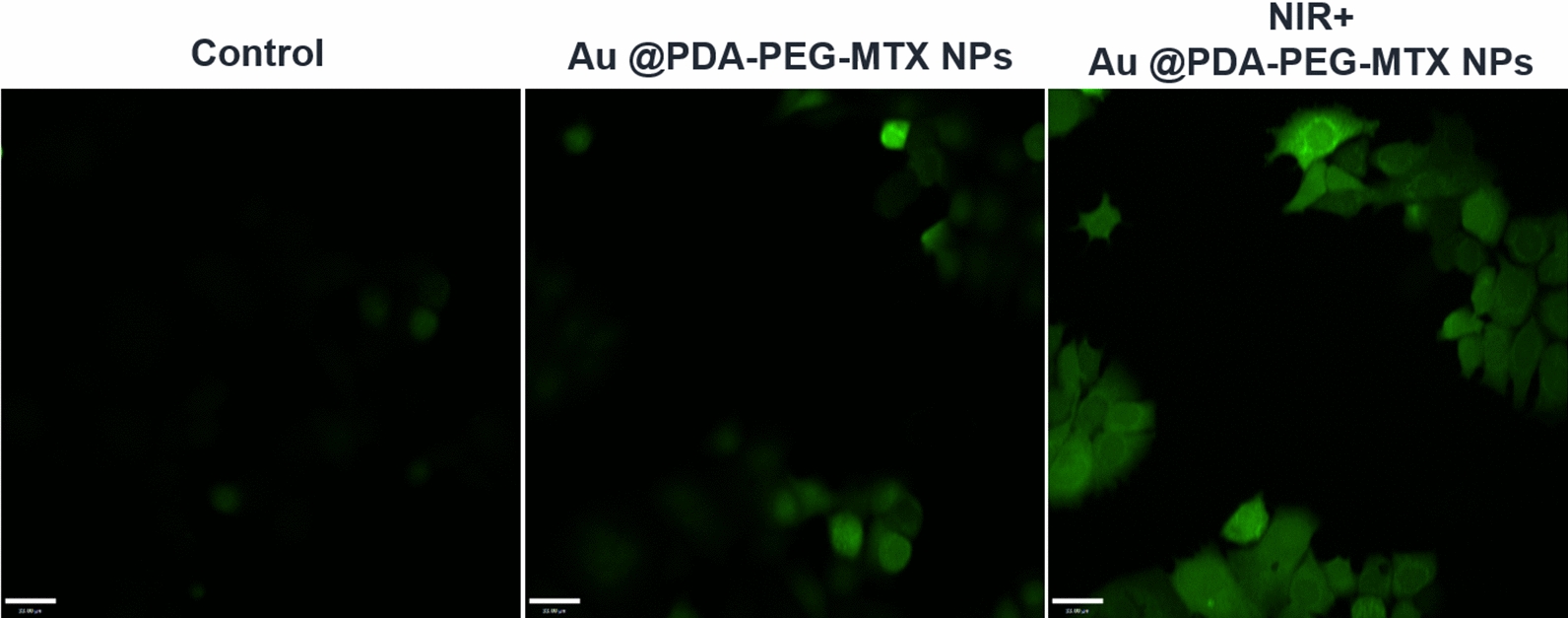
ROS generation in MDA-MB-231 cells with blank, Au @PDA-PEG-MTX and NIR + Au @PDA-PEG-MTX. ROS generation was detected by DCFH-DA probe

As shown in Fig. 7, the green fluorescence of Au @PDA-PEG-MTX NPs under NIR irradiation was enhanced compared to that of the blank cells in the control group and Au @PDA-PEG-MTX NPs. The results showed that Au @PDA-PEG-MTX NPs under NIR irradiation caused overproduction of ROS.

### Expression of apoptotic-related proteins in MDA-MB-231 cells

**Fig. 8:**
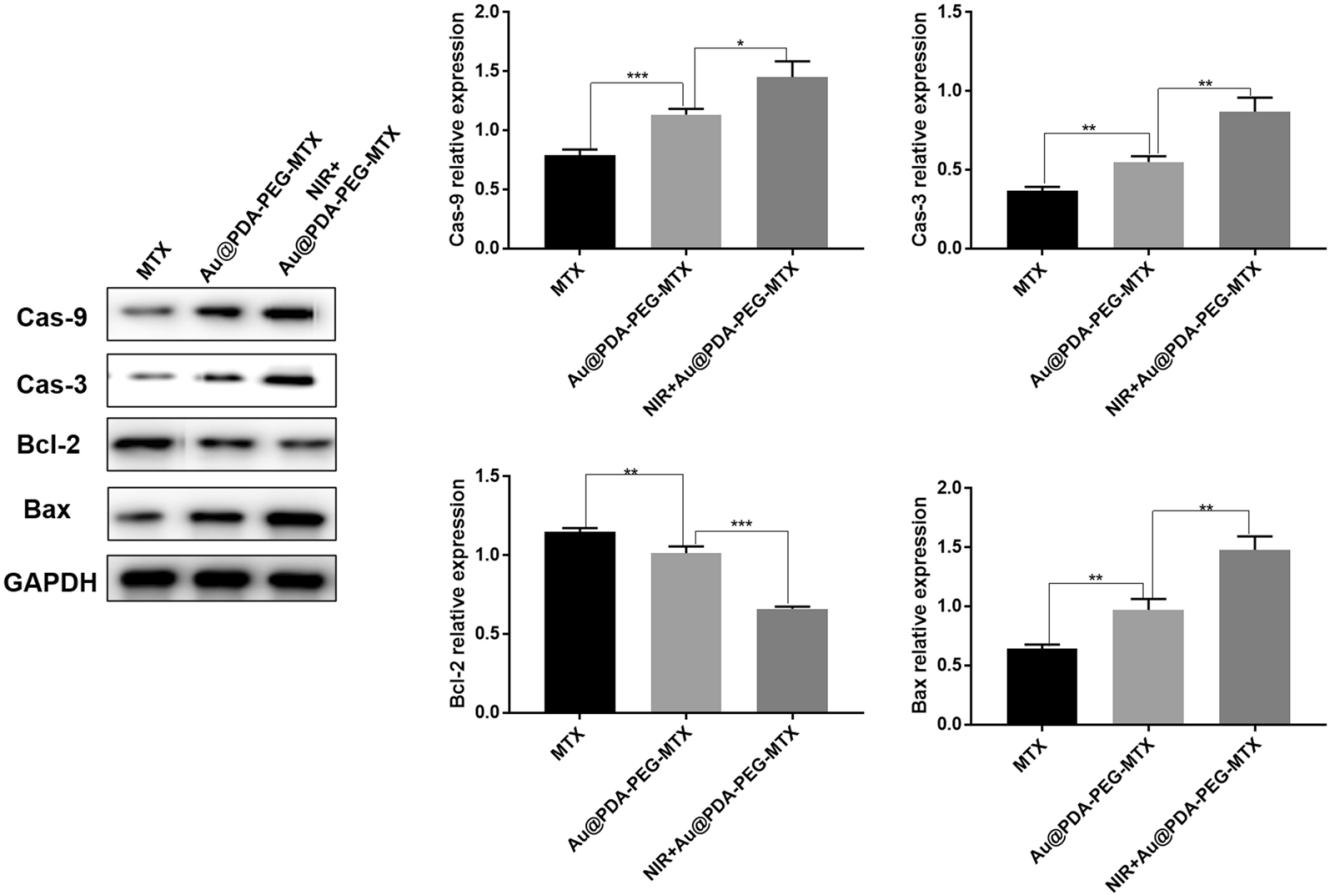
Expression of Cas-9, Cas-3, Bal-2 and Bax protein in MTX MDA-MB-231 cells with MTX, Au @PDA-PEG-MTX and NIR + Au @PDA-PEG-MTX (*p < 0.05, **p < 0.01)

Apoptosis referred to the orderly and autonomous death of cells controlled by genes in order to maintain a stable internal environment [38]. Mitochondria were the control centers for cell life activities, including apoptosis regulation along with cell respiratory chain and oxidative phosphorylation. Caspase played an essential role in the process of apoptosis [39]. It was reported that the release of cytochrome C from mitochondria was a key step in cell apoptosis. Cytochrome C released into the cytoplasm promoted the activation of Caspase-9, and then activated Caspase-9 which activated the downstream Caspase-3, inducing cell apoptosis [40]. Figure 8 showed the protein expression trends of cells treated with Au @PDA-PEG-MTX NPs with NIR laser irradiation, Au @PDA-PEG-MTX NPs alone and MTX alone. An increase in the expression of Caspase-3 and Caspase-9 was observed in MDA-MB-231 cells following treatment with NIR + Au @PDA-PEG-MTX NPs group compared with Au @PDA-PEG-MTX NPs alone or MTX alone groups.

The Bcl-2 family played the main role in anti-apoptosis. They were key regulators of the mitochondrial pathway. Overexpression of anti-apoptotic Bcl-2 and decreased expression of pro-apoptotic Bax were common in many human cancers. Bax was the first member of the pro-apoptotic family, and it was mainly located in the cytoplasm of normal cells. Bax was upregulated after being stimulated by apoptosis and transferred to the mitochondria, directly or indirectly, through formation of pores, causing the release of cytochrome C. Simultaneously, the expression of the anti-apoptotic protein Bcl-2 was downregulated. As shown in Fig. 8, the expression of Bax was increased and that of Bcl-2 decreased in NIR + Au @PDA-PEG-MTX NPs group, compared with Au @PDA-PEG-MTX NPs and MTX alone groups. In summary, NIR + Au @PDA-PEG-MTX NPs exhibited better therapeutic potential against BC.

### In vivo distribution and anticancer activity

**Fig. 9:**
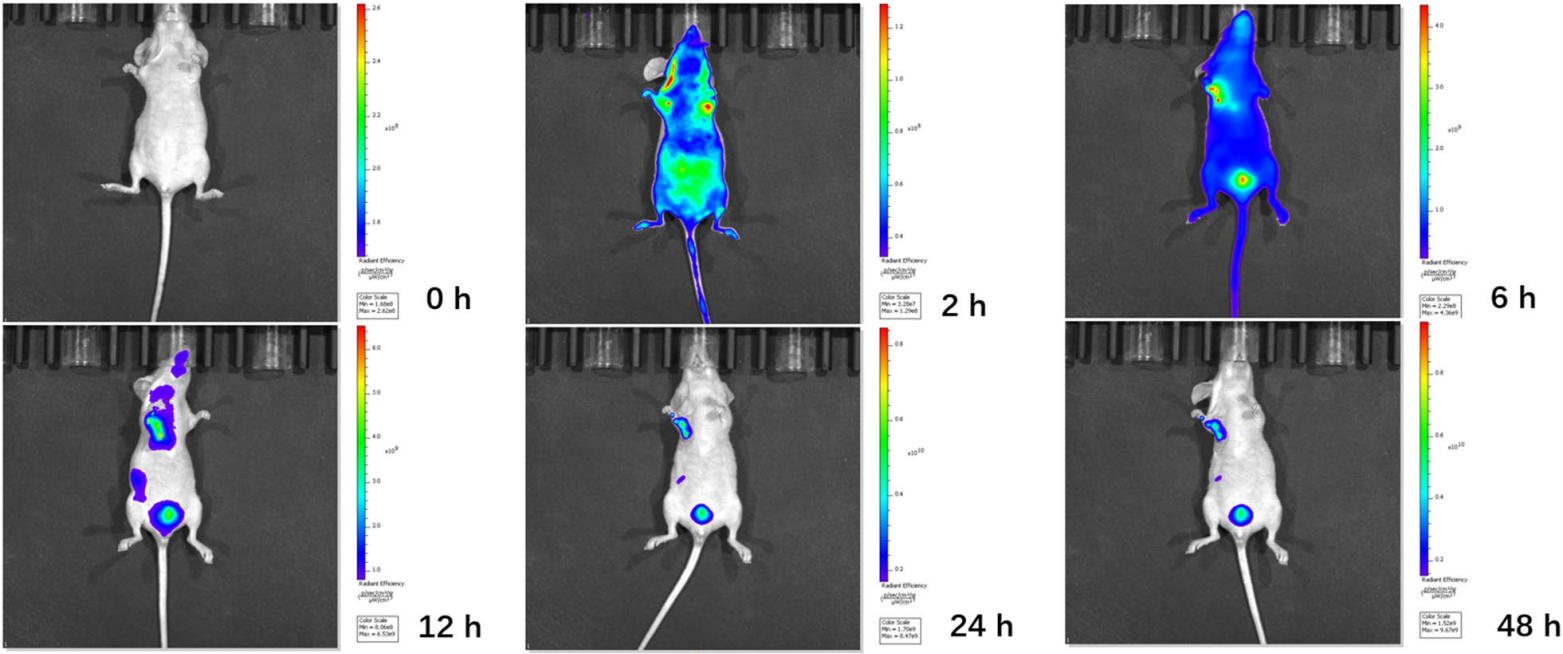
Real-time fluorescence imaging in vivo. In vivo bioluminescence imaging of the mice was examined at 0, 2, 4, 6, 12, 24 and 48 h after injection of Au @PDA/Cy7-PEG-MTX NPs into the body through the tail vein

As shown in Fig. 9, after injection of Au @PDA/Cy7-PEG-MTX NPs and Cy7 into the body through the tail vein within 2 h, the mice exposed to IVIS showed obvious biofluorescence in the tumor and/or surrounding areas. Compared with the NPs group, the Cy7 control group did not appear to be targeted to the tumor within 48 h (Additional file 1: Figure S1). For NPs groups, the fluorescent signal was detected in the tumor area after 6 h, and the complete aggregation to the tumor site was finally completed at 12 h, which confirmed the tumor-targeted imaging of Au @PDA/Cy7-PEG-MTX NPs in vivo. Then, Au @PDA/Cy7-PEG-MTX NPs were gradually metabolized and took effect in the body in 24–48 h.

In vivo anticancer activity was evaluated in BALB/c nude mice bearing MDA-MB-231 cancer xenografts. While BC tumor volume in mice of the control group (normal saline) was increased by approximately 15 times. After 19 days of treatment with MTX alone, the tumor weight was reduced by 44.68%. The reduction in tumor weight after treatment with NIR + Au @PDA-PEG-MTX NPs was 70.21%, which was markedly higher than that with MTX alone.

**Fig. 10:**
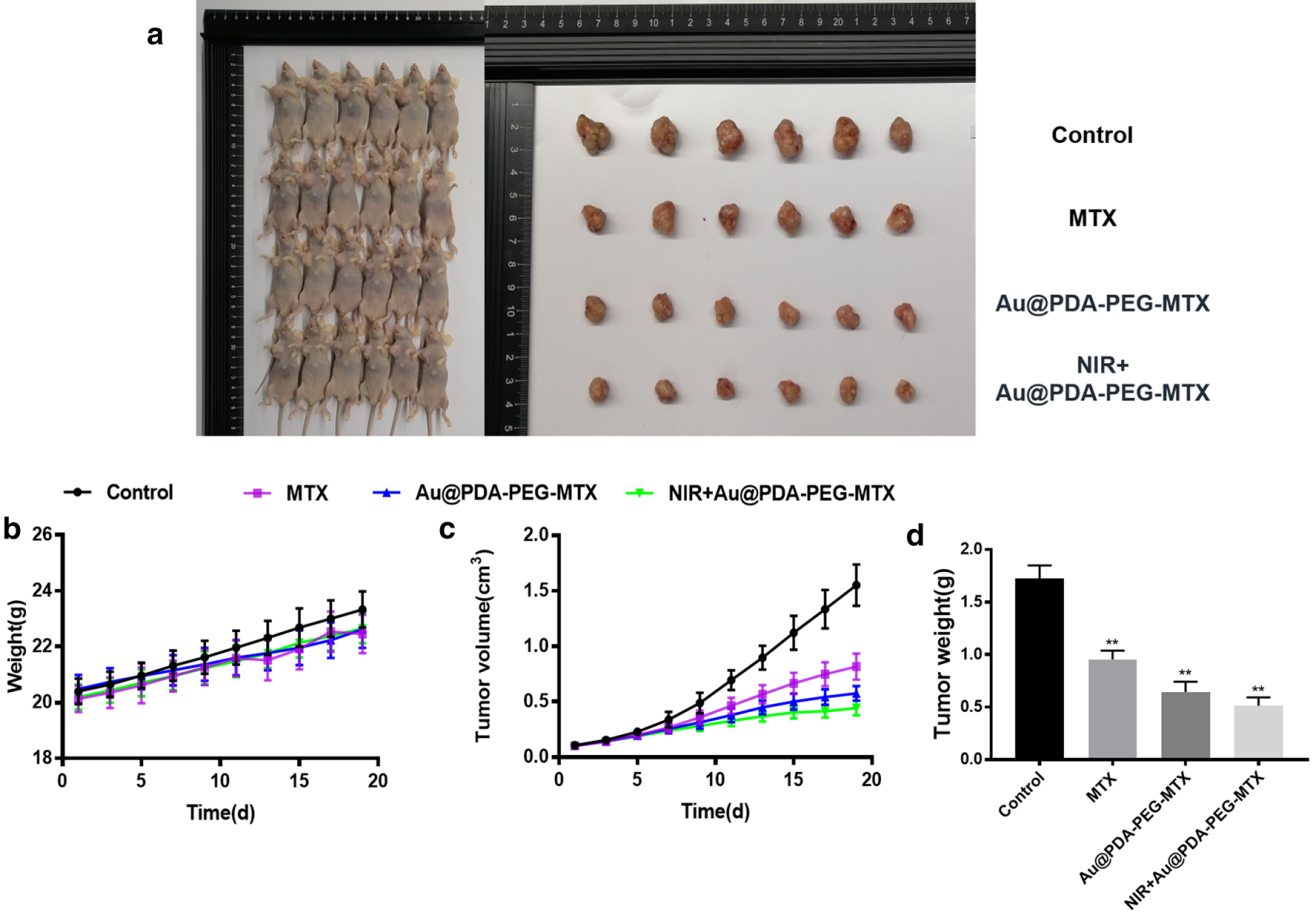
In vivo therapeutic effects. **a** Images of tumors; **b** Changes of BALB/c nude mice weight; **c** Changes of tumor volume; **d** Tumor weight measured in the nineteenth day (*p < 0.05, **p < 0.01)

Images of BALB/c nude mice and BC solid tumors were shown in Fig. 10a. The results showed that tail vein injection of MTX alone could only slightly suppress the volume of BC tumors. However, it was significantly suppressed after treatment with a combination of Au @PDA-PEG-MTX NPs with NIR. Statistical analysis of BC tumor volume (Fig. 10b), body weight changes in mice (Fig. 10c) and BC tumor weight (Fig. 10d) further confirmed the results of in vivo anticancer activity.

### In vitro safety evaluation

**Fig. 11:**
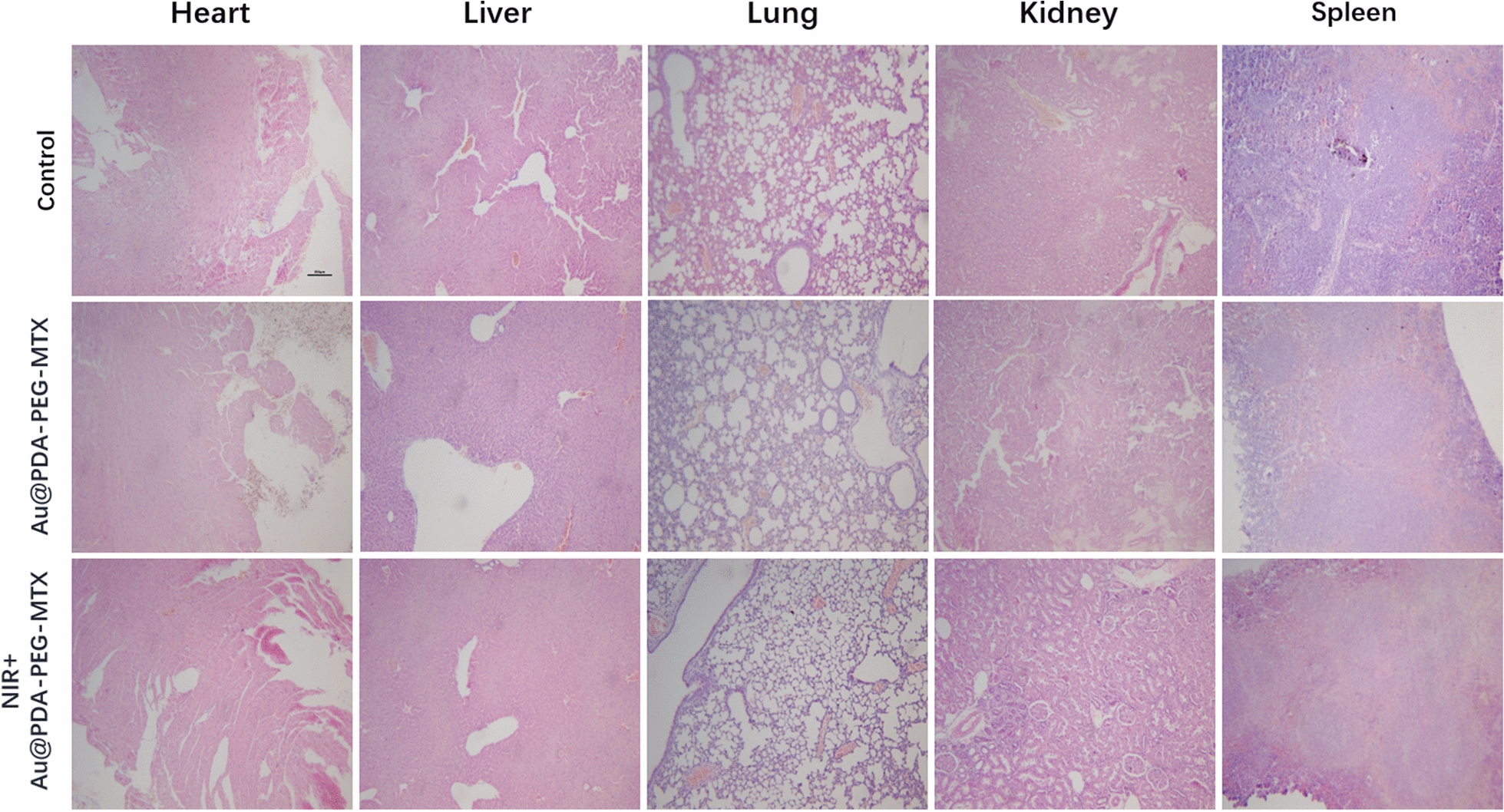
Histological analyses by H&E staining of heart, liver, spleen, lung and kidney in BALB/c mice that were treated with 0.9% NaCl, Au @PDA-PEG-MTX and NIR + Au @PDA-PEG-MTX

Side effects of Au @PDA-PEG-MTX NPs and NIR + Au @PDA-PEG-MTX NPs were examined to evaluate their safety. H&E staining was performed to observe histopathological changes. As shown in Fig. 11, cardiomyocytes were arranged regularly in the control group, with abundant cytoplasm, intact membrane and clearly visible nucleus, with no or occasional inflammatory cell infiltration. Au @PDA-PEG-MTX NPs and NIR + Au @PDA-PEG-MTX NPs groups were similar to the control group. In the control group, the number of hepatocytes was very abundant, the hepatic cords were regularly arranged, clear and complete, without obvious abnormalities; the blood vessels were round and there was little inflammation around the liver, and the liver lobules were rarely inflamed. In Au @PDA-PEG-MTX NPs group, hepatocytes were slightly swollen and deformed, hepatocytes had many fat droplets, liver lobules were degenerated, liver cords were arranged irregularly, and liver lobules were inflamed. Interestingly, histopathological findings of liver in Au @PDA-PEG-MTX NPs and NIR + Au @PDA-PEG-MTX NPs group were similar to those of the control group. In the control group, the glomerulus volume was approximately normal, the size was relatively uniform, the glomerular basement membrane was almost intact, the tubular epithelial cells were arranged regularly, and the tubular and interstitial-interstitial structures were acceptable. The structural characteristics of the two experimental groups were similar to those of the control group. In the control group and the two experimental groups, the lung tissue structure was relatively clear, the entire alveolar structure was relatively complete, the thickness of the alveolar wall was relatively normal, and the degree of bronchial stenosis was relatively light. Alveolar epithelial cells, eosinophils and lymphocytes rarely infiltrated the alveolar cavity, and the congestion sites in the alveoli and alveolar septum were significantly reduced. In the control group, the spleen had a clear structure, red and white pulps were regularly distributed, and no sinus congestion was seen. The structure of the splenic nodules was also observed as clear. Additionally, the structural characteristics of two experimental groups were similar to those of the control group.

These results indicated that Au @PDA-PEG-MTX NPs group and NIR + Au @PDA-PEG-MTX NPs had similar histological characteristics compared with the control group. It was indicated that Au @PDA-PEG-MTX NPs and NIR + Au @PDA-PEG-MTX NPs was basically non-toxic.

## Conclusions

A novel nanoplatform based on hierarchical drug release for chemo-photothermal treatment of BC was designed, which showed excellent anti-tumor efficacy and low toxicity. As a drug carrier, AuNPs showed good biocompatibility, stability and drug release triggered by NIR laser irradiation/pH. When Au @PDA-PEG-MTX NPs were taken up by tumor cells, MTX was released through amide bond cleavage in the specific acidic microenvironment (pH 5.5) of the lysosome as the first step of chemotherapy. Subsequently, NIR laser irradiation caused AuNPs to generate heat, and thereby MTX adsorbed on the surface of dopamine was released in the second step. The synthesized drug-loaded nanocarrier generated heat as well as a large amount of ROS, realizing multiple therapies, including chemo, photothermal and photodynamic therapies, to treat BC. The synthesized NPs possessed longer circulation time and targeted delivery of the drug, accompanied by bioimaging. We believe that the combination of multiple therapies to treat cancer is a promising strategy that will accelerate further developments in the field of oncology. In future research, detailed mechanisms in the delivery process and action forms will be pursued in depth for clinical purposes.

## Supplementary Information

**Additional file  1: Figure S 1. **Real-time fluorescence imaging in vivo. In vivo bioluminescence imaging of the mice was examined at 0, 2, 4, 6, 12, 24 and 48 hours after injection of Cy7 into the body through the tail vein.

## Data Availability

All data generated or analyzed during this study are included in this published article and supplementary information files.

## References

[1] Adak A, Unal YC, Yucel S, Vural Z, Turan FB, Yalcin-Ozuysal O, Ozcivici E, Mese G (2020). Connexin 32 induces pro-tumorigenic features in MCF10A normal breast cells and MDA-MB-231 metastatic breast cancer cells. Biochim Biophys Acta Mol Cell Res.

[2] Xiong K, Zhang Y, Wen Q, Luo J, Lu Y, Wu Z, Wang B, Chen Y, Zhao L, Fu S (2020). Co-delivery of paclitaxel and curcumin by biodegradable polymeric nanoparticles for breast cancer chemotherapy. Int J Pharm.

[3] Kim J, Shim M, Yang S, Moon Y, Song S, Choi J, Kim J, Kim K (2020). Combination of cancer-specific prodrug nanoparticle with Bcl-2 inhibitor to overcome acquired drug resistance. J Control Release..

[4] Venetis K, Invernizzi M, Sajjadi E, Curigliano G, Fusco N (2020). Cellular immunotherapy in breast cancer: The quest for consistent biomarkers. Cancer Treat Rev.

[5] Nunnery SE, Mayer IA (2020). Targeting the PI3K/AKT/mTOR pathway in hormone-positive breast cancer. Drugs..

[6] Tabassam Q, Mehmood T, Raza A, Ullah A, Saeed F, Anjum F (2020). Synthesis, characterization and anti-cancer therapeutic potential of with anolide-A with 20 nm sAuNPs conjugates against SKBR3 breast cancer cell line. Int J Nanomed.

[7] Zhu Y, Yang L, Xu J, Yang X, Luan P, Cui Q, Zhang P, Wang F, Li R, Ding X (2020). Discovery of the anti-angiogenesis effect of eltrombopag in breast cancer through targeting of HuR protein. Acta pharmaceutica Sinica B.

[8] He H, Liu L, Zhang S, Zheng M, Ma A, Chen Z, Pan H, Zhou H, Liang R, Cai L (2020). Smart gold nanocages for mild heat-triggered drug release and breaking chemoresistance. J Control Release.

[9] Wang W, Li D, Zhang Y, Zhang W, Ma P, Wang X, Song D, Sun Y (2020). One-pot synthesis of hyaluronic acid-coated gold nanoparticles as SERS substrate for the determination of hyaluronidase activity. Mikrochimica Acta.

[10] Bhatia E, Banerjee R (2020). Hybrid silver-gold nanoparticles suppress drug resistant polymicrobial biofilm formation and intracellular infection. J Mater Chem B.

[11] Bai X, Wang Y, Song Z, Feng Y, Chen Y, Zhang D, Feng L (2020). The basic properties of gold nanoparticles and their applications in tumor diagnosis and treatment. Int J Mol Sci..

[12] Chandrasekaran R, Madheswaran T, Tharmalingam N, Bose R, Park H, Ha D (2020). Labeling and tracking cells with gold nanoparticles. Drug Discov Today..

[13] Wang J, Zhang Y, Jin N, Mao C, Yang M (2019). Protein-induced gold nanoparticle assembly for improving the photothermal effect in cancer therapy. ACS Appl Mater Interfaces.

[14] Zheng T, Wang W, Wu F, Zhang M, Shen J, Sun Y (2019). Zwitterionic polymer-gated Au@TiO2 core-shell nanoparticles for imaging-guided combined cancer therapy. Theranostics.

[15] Hou Z, Wang Z, Liu R, Li H, Zhang Z, Su T, Yang J, Liu H (2019). The effect of phospho-peptide on the stability of gold nanoparticles and drug delivery. J Nanobiotechnol..

[16] Amouzadeh Tabrizi M, Shamsipur M, Saber R, Sarkar S (2018). Isolation of HL-60 cancer cells from the human serum sample using MnO2-PEI/Ni/Au/aptamer as a novel nanomotor and electrochemical determination of thereof by aptamer/gold nanoparticles-poly(3,4-ethylene dioxythiophene) modified GC electrode. Biosens Bioelectron.

[17] Malaikolundhan H, Mookkan G, Krishnamoorthi G, Matheswaran N, Alsawalha M, Veeraraghavan V, Krishna Mohan S, Di A (2020). Albizia lebbeck anticarcinogenic effect of gold nanoparticles synthesized from on HCT-116 colon cancer cell lines. Artif Cells Nanomed Biotechnol.

[18] Zheng Y, Zhang J, Zhang R, Luo Z, Wang C, Shi S (2019). Gold nano particles synthesized from Magnolia officinalis and anticancer activity in A549 lung cancer cells. Artif Cells Nanomed Biotechnol.

[19] Scarano S, Palladino P, Pascale E, Brittoli A, Minunni M (2019). Colorimetric determination of p-nitrophenol by using ELISA microwells modified with an adhesive polydopamine nanofilm containing catalytically active gold nanoparticles. Mikrochim Acta.

[20] Sy KHS, Ho LWC, Lau WCY, Ko H, Choi CHJ (2018). Morphological diversity, protein adsorption, and cellular uptake of polydopamine-coated gold nanoparticles. Langmuir.

[21] Cai S, Yan J, Xiong H, Xing H, Liu Y, Liu S, Liu Z (2020). Aptamer-functionalized molybdenum disulfide nanosheets for tumor cell targeting and lysosomal acidic environment/NIR laser responsive drug delivery to realize synergetic chemo-photothermal therapeutic effects. Int J Pharm.

[22] Mao W, Kim HS, Son YJ, Kim SR, Yoo HS (2018). Doxorubicin encapsulated clicked gold nanoparticle clusters exhibiting tumor-specific disassembly for enhanced tumor localization and computerized tomographic imaging. J Control Release.

[23] Feito MJ, Diez-Orejas R, Cicuendez M, Casarrubios L, Rojo JM, Portoles MT (2019). Characterization of M1 and M2 polarization phenotypes in peritoneal macrophages after treatment with graphene oxide nanosheets. Colloids Surf B Biointerfaces.

[24] Liu R, An Y, Jia W, Wang Y, Wu Y, Zhen Y, Cao J, Gao H (2020). Macrophage-mimic shape changeable nanomedicine retained in tumor for multimodal therapy of breast cancer. J Controlled Release.

[25] He Y, Cong C, Li X, Zhu R, Li A, Zhao S, Li X, Cheng X, Yang M, Gao D (2019). Nano-drug system based on hierarchical drug release for deep localized/systematic cascade tumor therapy stimulating antitumor immune responses. Theranostics.

[26] Murawala P, Tirmale A, Shiras A, Prasad BL (2014). In situ synthesized BSA capped gold nanoparticles: effective carrier of anticancer drug methotrexate to MCF-7 breast cancer cells. Mater Sci Eng C Mater Biol Appl.

[27] Ong Y, Bañobre-López M, Costa Lima S, Reis S (2020). A multifunctional nanomedicine platform for co-delivery of methotrexate and mild hyperthermia towards breast cancer therapy. Materials science engineering C Materials for biological applications.

[28] Ong YS, Banobre-Lopez M, Costa Lima SA, Reis S (2020). A multifunctional nanomedicine platform for co-delivery of methotrexate and mild hyperthermia towards breast cancer therapy. Mater Sci Eng C Mater Biol Appl.

[29] Ali EMM, Elashkar AA, El-Kassas HY, Salim EI (2018). Methotrexate loaded on magnetite iron nanoparticles coated with chitosan: Biosynthesis, characterization, and impact on human breast cancer MCF-7 cell line. Int J Biol Macromol.

[30] Dutta B, Nema A, Shetake NG, Gupta J, Barick KC, Lawande MA, Pandey BN, Priyadarsini IK, Hassan PA (2020). Glutamic acid-coated Fe3O4 nanoparticles for tumor-targeted imaging and therapeutics. Mater Sci Eng C Mater Biol Appl.

[31] Chen TW, Jan IS, Chang DY, Lin CH, Chen IC, Chen HM, Cheng AL, Lu YS (2020). Systemic treatment of breast cancer with leptomeningeal metastases using bevacizumab, etoposide and cisplatin (BEEP regimen) significantly improves overall survival. J Neurooncol.

[32] Wang C, Vazquez-Gonzalez M, Fadeev M, Sohn YS, Nechushtai R, Willner I (2020). Thermoplasmonic-triggered release of loads from DNA-modified hydrogel microcapsules functionalized with Au nanoparticles or Au nanorods. Small.

[33] Zhang X, Feng Y, Duan S, Su L, Zhang J, He F (2019). Mycobacterium tuberculosis strain H37Rv Electrochemical Sensor Mediated by Aptamer and AuNPs-DNA. ACS Sens.

[34] Liu P, Wang Y, Liu Y, Tan F, Li J, Li N (2020). S-nitrosothiols loaded mini-sized Au@silica nanorod elicits collagen depletion and mitochondrial damage in solid tumor treatment. Theranostics.

[35] Sargazi A, Kamali N, Shiri F, Heidari Majd M (2018). Hyaluronic acid/polyethylene glycol nanoparticles for controlled delivery of mitoxantrone. Artif Cells Nanomed Biotechnol.

[36] Wu C, Tong Y, Wang P, Wang D, Wu S, Zhang J (2013). Identification of impurities in methotrexate drug substances using high-performance liquid chromatography coupled with a photodiode array detector and Fourier transform ion cyclotron resonance mass spectrometry. Rapid Commun Mass Spectr.

[37] De AK, Muthiyan R, Mondal S, Mahanta N, Bhattacharya D, Ponraj P, Muniswamy K, Kundu A, Kundu MS, Sunder J (2019). A natural quinazoline derivative from marine sponge hyrtios erectus induces apoptosis of breast cancer cells via ROS production and intrinsic or extrinsic apoptosis pathways. Mar Drugs..

[38] Zhang Y, Hai Y, Miao Y, Qi X, Xue W, Luo Y, Fan H, Yue T (2020). The toxicity mechanism of different sized iron nanoparticles on human breast cancer (MCF7) cells. Food Chem.

[39] Szoka L, Palka J (2020). Capsaicin up-regulates pro-apoptotic activity of thiazolidinediones in glioblastoma cell line. Biomed Pharmacother.

[40] Liu X, Ma R, Yi B, Riker A, Xi Y (2020). MicroRNAs are involved in the development and progression of gastric cancer. Acta Pharmacol Sinica..

