# Supplementary material for: Hierarchical drug release designed Au @PDA-PEG-MTX NPs for targeted delivery to breast cancer with combined photothermal-chemotherapy
Source: J Nanobiotechnology. 2021 May 17;19:143. doi: 10.1186/s12951-021-00883-8 (PMC8130275; doi:10.1186/s12951-021-00883-8)
Supplement: Supplementary file 1 — Additional file 1: Figure S 1. Real-time fluorescence imaging in vivo. In vivo bioluminescence imaging of the mice was examined at 0, 2, 4, 6, 12, 24 and 48 hours after injection of Cy7 into the body through the tail vein. [file 12951_2021_883_MOESM1_ESM.docx]

Supporting Information

**Hierarchical Drug Release Designed Au @PDA-PEG-MTX NPs for Targeted Delivery to Breast Cancer with Combined Photothermal-chemotherapy**

Wen Li^1^, Zhiwen Cao^1^, Liuchunyang Yu^1^, Qingcai Huang^1^, Dongjie Zhu^1^, Cheng Lu^2*^, Aiping Lu^3*^, Yuanyan Liu^1*^

1 School of Chinese Materia Medica, Beijing University of Chinese Medicine, Beijing 100029, China

2 Institute of Basic Research in Clinical Medicine, China Academy of Chinese Medical Sciences, Beijing 100700, China

3 School of Chinese Medicine, Hong Kong Baptist University, Kowloon, Hongkong, China

* Corresponding Author

Dr. Yuanyan Liu: [yyliu_1980@163.com](mailto:yyliu_1980@163.com). Tel: +86 10 84738658.

Dr. Cheng Lu: lv_cheng0816@163.com.

Dr. Aiping Lu: [lap64067611@126.com](mailto:lap64067611@126.com).


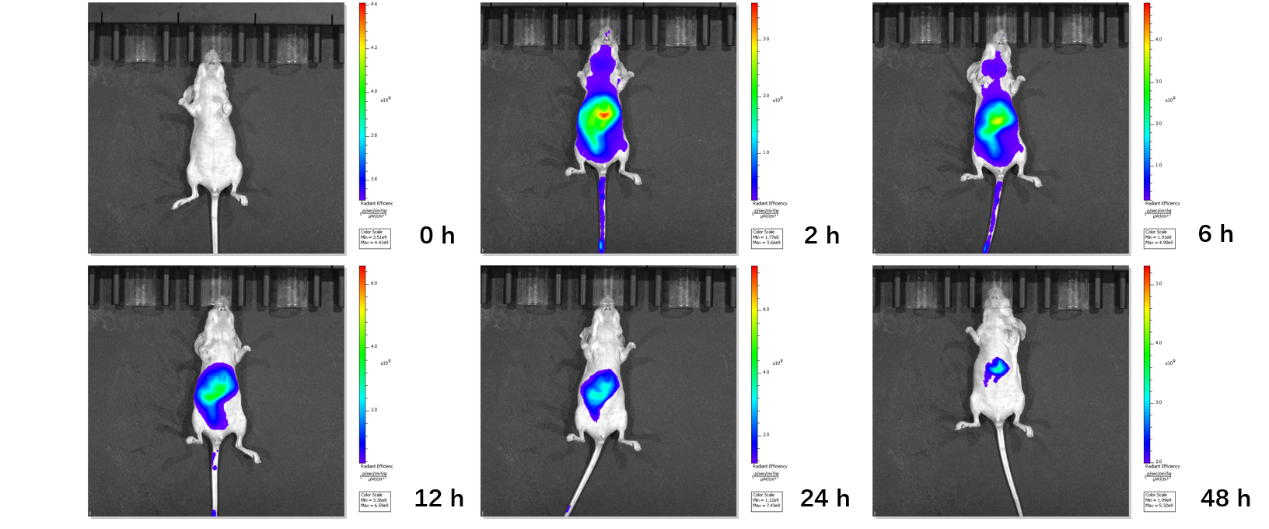


Figure S 1. Real-time fluorescence imaging in vivo. In vivo bioluminescence imaging of the mice was examined at 0, 2, 4, 6, 12, 24 and 48 hours after injection of Cy7 into the body through the tail vein.
